# Cutting-edge AI tools revolutionizing scientific research in life sciences

**DOI:** 10.5114/bta/200803

**Published:** 2025-03-31

**Authors:** Katarzyna Lorenc-Kukuła

**Affiliations:** Translmed Publishing Group, Wieluñ, Poland

**Keywords:** deep learning, machine learning, AI-driven discovery, predictive modeling, artificial intelligence, 2024 Nobel prizes

## Abstract

Artificial intelligence (AI) is becoming a transformative force in the life sciences, pushing the boundaries of possibility. Imagine AI automating time-consuming tasks, uncovering hidden patterns in vast datasets, designing proteins in minutes instead of years, and even predicting disease outbreaks before they occur. This review explores the latest AI tools revolutionizing scientific fields, including research and data analysis, healthcare, and tools supporting scientific writing. Beyond data processing, AI is reshaping how scientists draft and share their findings, enhancing processes ranging from literature reviews to citation management. However, with great power comes great responsibility. Are we prepared for this leap? This review delves into the forefront of AI in the life sciences, where innovation meets responsibility.

## Introduction

**Figure 1:**
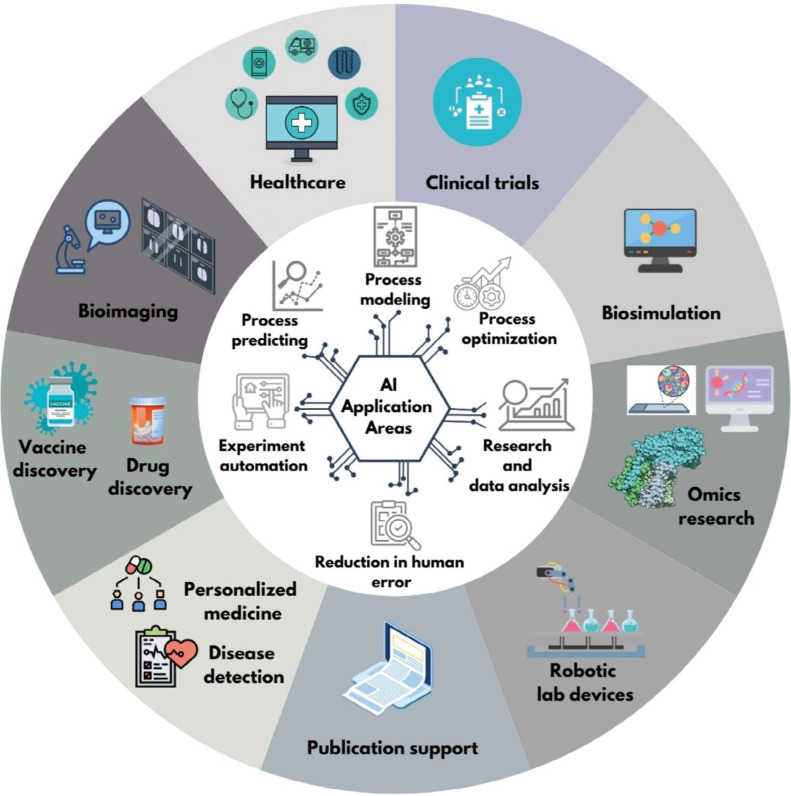
Applications of AI in scientific research

Artificial intelligence (AI) is revolutionizing scientific research, particularly in the field of life sciences. AI advances science by enabling the analysis of data and addressing challenges that were previously beyond the scope of traditional research methods. Tools such as **AlphaFold**, which has revolutionized structural biology and contributed to a Nobel Prize-winning breakthrough, and **BioBERT**, a natural language processing model for biomedical text analysis, are prime examples of how AI supports scientific research. AI tools assist in analyzing large datasets, automating repetitive tasks, predicting, optimization and modeling complex processes, thereby accelerating scientific discovery. Figure 1 illustrates the application possibilities of artificial intelligence in the fields of healthcare, clinical trials, biosimulation, omics research, personalized medicine, early disease detection, vaccine and drug discovery, bioimaging, robotic laboratory equipment, and streamlining scientific paper writing.

Below, an overview of the latest AI tools and their potential applications in life sciences is presented, along with tools that aid scientists in writing scientific publications.

## AI in proteomics

AI plays a transformative role in proteomics by revolutionizing the prediction of protein structures. Tools like **AlphaFold**, developed by DeepMind, predict protein structures with unprecedented accuracy based solely on amino acid sequences. This breakthrough has significantly accelerated research on protein functions and their roles in diseases, facilitating drug development and disease research. The significance of AI in this field was underscored in 2024 when the Nobel Prize in Chemistry was awarded to three pioneers for their development of AI-based methods for predicting protein structures (Callaway, 2024). Demis Hassabis and John Jumper of Google DeepMind were recognized for their AI programs that accurately predict the 3D shapes of proteins, while David Baker from the University of Washington was honored for his work in designing novel proteins. These advancements are essential for understanding biomolecular functions and for developing drugs and vaccines (Protein designer and structure solvers, 2024; Royal Swedish Academy of Sciences, 2024).

Previously, imaging techniques such as X-ray crystallography and cryo-electron microscopy were the primary methods for determining protein structures. However, these methods were time-consuming, expensive, and often challenging to apply to all proteins. In 2020, the introduction of **AlphaFold 2** revolutionized protein structure prediction. By leveraging vast databases of protein structures and amino acid sequences, it achieved performance comparable to imaging methods and delivered over 200 million predictions (Service, 2020). In 2021, DeepMind launched Isomorphic Labs to lever-age its AI tools for drug design, inspiring pharmaceutical companies worldwide to adopt similar approaches to combat cancer, infectious diseases, hypertension, and obesity (Protein designer and structure solvers, 2024). Within just 3 years, AlphaFold 2 enabled 1.8 million researchers to map approximately six million different protein structures. In a groundbreaking development, DeepMind recently released **AlphaFold 3**, which provides results in minutes instead of years of laboratory work (Abramson et al., 2024). Unlike earlier versions, which focused on modeling amino acid strands folding into 3D protein shapes, AlphaFold 3 also predicts how folded proteins bind and interact with other molecules, including DNA, RNA, and other proteins. AlphaFold 3 was released shortly after **RoseTTAFold All-Atom** (Krishna et al., 2024), a similar AI tool from the University of Washington led by David Baker. To promote widespread adoption, DeepMind introduced AlphaFold Server, a free online platform allowing users to generate AlphaFold 3 models of proteins interacting with nearly any biomolecule (AlphaFold Server, 2024).

Understanding interactions between proteins and other biological molecules is essential for drug design and the study of cellular processes. **AlphaFold**’s breakthroughs in protein structure prediction are critical for understanding protein functions, with profound implications for drug development, biotechnology, and disease research. These advancements highlight the transformative role of AI in advancing proteomics and biomedical research. **AlphaFold** has ushered in a new era in science and medicine, revolutionizing our understanding of protein interactions and enabling groundbreaking advancements in drug development and biotechnology.

## AI in genomic research

In biological research, especially in genomics, proteomics, metabolomics, and transcriptomics, vast quantities of data are generated. AI has become a cornerstone in clinical laboratory genomics, assisting in tasks such as identifying variants in DNA sequencing data, predicting the effects of DNA variants on protein structure and function, linking phenotype ontologies to genetic variants for faster diagnosis, correlating genomic data with tumor staging and treatment, utilizing natural language processing (NLP) to identify critical medical literature, and using interactive chatbots for genetic testing qualification and education (Aradhya et al., 2023). AI addresses challenges in genomic data analysis arising from high-throughput sequencing (Boulesteix and Wright, 2022). It facilitates GWAS and PheWAS to identify genotype-phenotype associations, improves pharmacogenomics, supports clinical decisions, predicts risks, identifies causal SNPs, enhances EHR-based phenotyping, and designs CRISPR guide RNA (Lin and Ngiam, 2023). Traditional analysis methods often fall short, prompting the adoption of AI, which enhances both the speed and accuracy of genomic data analysis. AI tools, including computer vision (CV), machine learning (ML), neural networks, and NLP, have become indispensable for addressing challenges in genomic data analysis (Guo et al., 2023). They assist in the analysis, interpretation, modeling, and processing of large-scale genomic data. These tools are utilized in genetic and genomic research across various diseases (Libbrecht and Noble, 2015; Yan, et al., 2016; Dias and Torkamani, 2019; Xu et al., 2019; Alimadadi et al., 2020; De Marvao et al., 2020), biomarker discovery studies (Seashore-Ludlow et al., 2015; Mamatjan et al., 2017), annotating genomic sequence elements (Libbrecht and Noble, 2015), predicting gene functions, understanding regulatory networks, and identifying disease-associated variants.

Recent studies increasingly focus on integrating machine learning techniques to process and analyze high-dimensional genomic data. AI is transforming biomedical genomics by improving data analysis, enhancing disease prediction, and advancing personalized medicine. The integration of AI techniques, such as machine learning and deep learning, allows for efficient processing of complex genomic data, leading to significant progress in biomarker discovery and genetic engineering. AI tools also automate labor-intensive processes, improve diagnostic precision, and facilitate the interpretation of complex genomic data, ultimately revolutionizing genomic research and its clinical applications. A comprehensive review in 2023 summarized 82 high-quality AI-driven biomedical genomic studies, emphasizing AI’s contributions to disease diagnosis, prediction, and treatment (Guo et al., 2023). The review highlighted various AI techniques, including **ML**, deep neural networks (**DNN**), transfer learning (**TL**), **CV**, graph representation learning (**GRL**), and **NLP**. In this manuscript, we briefly discuss each of the AI techniques mentioned above:

- **ML** is widely used for analyzing large genomic datasets and identifying patterns and relationships within the data. ML encompasses techniques such as linear regression, logistic regression, decision trees, random forests (RF), support vector machines (SVMs), and neural networks (Guo et al., 2023). These methods are indispensable in medicine, particularly for diagnosing SARS-CoV-2 infections, neurological conditions (Deo, 2015; Rajkomar et al., 2019; Sidey-Gibbons and Sidey-Gibbons, 2019; Helmy et al., 2022), identifying genes linked to sepsis-induced ARDS, pinpointing chronic pain locations, uncovering genetic risk factors for various conditions, and diagnosing cancers such as leukemia, breast and lung cancer, and endometrial carcinoma (Guo et al., 2023).
- Convolutional neural networks **(CNNs)** are a prominent type of **DNN**. They have demonstrated significant effectiveness in identifying cancers by analyzing gene expression profiles. In one study, researchers analyzed 6136 samples from 11 cancer types, integrating gene expression profiles with protein–protein interaction (PPI) networks to create 2D images (Chuang et al., 2021). These images were used to develop a CNN that achieved high accuracy in distinguishing normal samples from tumors and identifying specific cancer types. CNNs are not only valuable for diagnosing and predicting cancer outcomes but also for identifying various cancer biomarkers. In the field of cancer research, DNN-based applications have been applied for detection purposes (Zhang et al., 2022). By utilizing medical images and omics data, CNN techniques were used to detect metastasis indicators, cancer cell types, and molecular subtypes using medical images and omics data, providing critical information essential for therapeutic management (Chuang et al., 2021).
- **Transfer learning** is a sophisticated AI approach in genomics particularly useful in cross-population studies (Zhao et al., 2022). It enhances predictive performance for disease risk (Jónsson et al., 2019), predicts patient data trends, and provides new insights into clinical findings from genotypic information (Dong and Boyle, 2019). Transfer learning also improves functional variant prediction accuracy, enhances enhancer-promoter interaction predictions, imputes missing RNAsequencing data, and predicts rare diseases using gene expression datasets (Guo et al., 2023). In healthcare, transfer learning boosts diagnostic accuracy for cancer detection, mutation identification, cancer type detection from circulating tumor cells, and studying circular RNAs in nonobstructive azoospermia (Guo et al., 2023).
- Another AI tool widely used in genomic analysis is **recurrent neural networks (RNNs)**. RNNs are particularly valuable for analyzing biomedical data collected over time. While CNNs excel in image analysis, they do not account for temporal dimensions. RNNs, designed for sequential data, are ideal for analyzing time-series data such as patient health records. By incorporating temporal aspects, RNNs enable the understanding of data progression and changes over time, which is crucial for accurate predictions and informed decisions in genomics (Guo et al., 2023). For instance, RNNs significantly enhanced genomic analysis for predicting chronic diseases, as demonstrated in a Type 2 diabetes study using UK Biobank data by Srinivasu et al. (2022).
- **CV** uses mathematical methods to derive three-dimensional shapes and appearances of objects from images. Popular architectures such as **AlexNet**, **VGGNet**, **GoogLeNet**, and **ResNet** have broad applications in biomedical genomic research, including linking imaging phenotypes to tumor genetic profiles (Bodalal et al., 2019).
- **GRL** is used to transform complex genomic data into simplified, low-dimensional vectors, facilitating easier analysis and interpretation. In genomics, GRL is particularly valuable for converting sequencing data into graph structures based on gene associations or expression similarities (Guo et al., 2023). Applications of GRL techniques include diagnosing diseases such as multiple sclerosis using graph attention networks on single-cell RNA sequencing data, classifying cancers with graph convolutional networks that integrate gene expression and protein–protein interaction data, and identifying cancer subtypes and intracluster heterogeneity using dimensionality reduction techniques. GRL also aids in identifying new candidate disease genes, leveraging gene–disease associations for clinical investigation, performing link prediction tasks, integrating heterogeneous associations, constructing networks with various gene nodes, and predicting gene–disease associations and disease classifications (Guo et al., 2023).
- In genomic research, **NLP** assists by converting textual data, such as Electronic Health Records (EHRs), into computable features for Genome-Wide Association Studies (GWAS) and Phenome-Wide Association Studies (PheWAS). It also extracts detailed phenotypic characteristics for diagnosing genetic disorders (Guo et al., 2023). **BioBERT**, a revolutionary NLP tool, has significantly advanced biomedical research (Lee et al., 2020). Pretrained on vast amounts of biomedical literature, BioBERT enables researchers to understand and interpret complex biomedical information, making it invaluable for biomedical text mining, drug discovery, disease research, and biotechnology (Lee et al., 2020). The rapid expansion of biomedical literature underscores the need for tools like BioBERT to handle the influx of information effectively. Its open-access model (Lee et al., 2020), which includes pre-trained weights and fine-tuning source code, allows researchers worldwide to leverage this technology without incurring significant costs.

The volume of data on AI applications in gene and genome analysis continues to grow. Recently, Hu et al. (2024) in *Nature Methods* highlighted the effectiveness of large language models (LLMs) such as **GPT-4**, **GPT-3.5**, **Gemini Pro**, **Mixtral Instruct**, and **Llama2 70b** in gene function analysis. These models demonstrated greater specificity and broader gene coverage compared to traditional methods, illustrating their potential to automate functional genomics research. LLMs like GPT-4 can facilitate the understanding of gene functions and interactions.

AI has proven instrumental in identifying complex structural variants (cxSVs) in whole-genome sequencing data. Researchers at Stanford Medicine have developed **ARC-SV**, an AI-based method leveraging machine learning to improve the precision of detecting and reconstructing cxSVs from standard datasets. This technique not only uncovers rare genetic variations but also connects them to neural genes and regions of rapid human-specific evolution. Furthermore, it links cxSVs to differences in gene expression and chromatin accessibility across various brain regions, advancing the genetic understanding of major psychiatric disorders (Zhou et al., 2024).

The advent of artificial intelligence has driven transformative breakthroughs in genetic medicine. Genomic medicine—the application of genomic information in clinical treatments—holds immense potential for personalized and targeted medical interventions. The integration of AI algorithms into genomic data processing has yielded remarkable successes. These algorithms excel at decoding complex genetic patterns, predicting disease probabilities, and enhancing precision medicine. A study by Hassan et al. (2022) in *Nature Medicine* demonstrated that machine learning algorithms can accurately predict patient responses to cancer immunotherapy based on genetic profiles. This finding underscores AI’s transformative impact on healthcare, particularly in advancing genomic medicine, providing insights into disease causation, and enabling personalized treatment plans. By leveraging advanced algorithms and large datasets, AI is poised to revolutionize healthcare, tailoring medical treatments to individual patients.

The pursuit of fast, affordable, and precise DNA sequencing remains a critical goal in advancing personalized medicine. Conventional bioinformatics methods often struggle to efficiently process and interpret the vast volumes of generated data. In contrast, deep neural networks (DNNs) excel at recognizing complex patterns, predicting phenotypes, and classifying genomic variants. Recent advancements in deep learning have demonstrated its effectiveness in various biomedical applications, especially in Next-Generation Sequencing (NGS). These methods employ advanced neural networks to process extensive genomic data, enhancing sequencing accuracy and efficiency. By automating data analysis and uncovering patterns within complex datasets, deep learning significantly advances our understanding of genetic variations and their health implications (Özgür and Orman, 2023). Integrating machine learning algorithms into NGS workflows has the potential to reveal hidden insights, accelerate discoveries, and drive breakthroughs in genomics.

AI can enhance our understanding of how single amino acid changes in proteins impact their function. The deep learning model **AlphaMissense**, building on the protein structure prediction tool **AlphaFold2**, utilizes vast biological sequence data and predicted structural contexts to assess the pathogenicity of gene variants. This capability is essential for understanding the implications of genetic variations in disease contexts (Cheng et al., 2023). AlphaMissense predictions play a pivotal role in elucidating how genetic variants affect protein function, aiding in the identification of pathogenic missense mutations and previously undetected diseasecausing genes. This advancement improves diagnostic accuracy for rare genetic disorders and fosters the development of advanced tools for predicting protein variant effects based on structural models.

In 2024, researchers introduced another therapeutic application of AI: designing and validating engineered cis-regulatory elements (**CREs**) using AI models. These CREs were tailored for targeted gene expression in specific cell types (Gosai et al., 2024). AI-engineered synthetic CREs demonstrated the potential to target gene therapies to particular cell populations. Deep neural network modeling was used to predict CRE activity across different cell types, while a high-throughput method called **Massively Parallel Reporter Assays (MPRAs)** enabled the rapid empirical testing of thousands of designed CREs. Results showed that synthetic CREs were more effective for targeted gene expression than natural sequences derived from the human genome, highlighting their utility in therapeutic and biotechnological applications. By leveraging AI in the design of synthetic CREs, scientists can create programmable regulatory elements with precise targeting capabilities, enhancing the efficacy and specificity of gene therapies (Gosai et al., 2024).

**PEACOCK** is another example of an AI tool used to analyze large datasets typical in genomic research, through machine learning techniques. This tool efficiently analyzed vast amounts of genomic data to identify potential regulatory links that may not be easily discernible through traditional methods. This model could predict cell type-specific enhancer-gene regulatory links and was trained using various cell lines and a selected set of experimental data validated and published in scientific literature. The efficiency and scalability of the PEACOCK were proved by its ability to score a vast number of enhancer–gene pairs across the entire genome (∼17 million pairs). The ability to score enhancer–gene pairs quantitatively allows researchers to incorporate these scores into broader statistical analyses of disease-associated variants. The scores generated by the PEACOCK model have significant implications for disease research, particularly in understanding the role of enhancers in gene regulation. The quantitative scores allow researchers to prioritize enhancer–gene pairs that are most likely to be involved in disease processes. By focusing on high-scoring pairs, researchers can concentrate on the most promising enhancer–gene pairs in diseases like cancer, can gain insights into the molecular mechanisms underlying diseases and hopefully lead to new therapeutic targets. Moreover, the cell typespecific nature of the scores can help understand how gene regulation may differ across various tissues, which is especially important in diseases where certain genes may be activated or silenced. These scores can be used in the statistical research approach of genomic disease-associated variants identified in a GWAS (Genome-Wide Association Studies, 2024). GWAS surveys the genomes of people, looking for genomic variants that occur more frequently in those with a specific disease compared to those without the disease. Once such genomic variants are identified, they are used to search for nearby variants that contribute directly to the disease. By linking these variants to specific enhancer–gene interactions, researchers can uncover how genetic variations contribute to disease risk and progression, potentially leading to new therapeutic targets. Understanding which enhancers are active or inactive in specific cell types can help us understand how disruption in enhancer function contributes to various diseases and genetic disorders. Scores designed by the PEACOCK model can serve as a useful tool for disease research by enabling targeted investigations, enhancing understanding of disease mechanisms, and linking genetic variants to regulatory relationships. This approach can contribute to the development of new treatments for disease prevention. The **PEREGRINE database** (**www.peregrineproj.org**), a publicly available resource was developed as part of the PEACOCK study. These resources allow researchers to access curated data on enhancer–gene links, facilitating further research and validation of findings in genomic studies (Mills et al., 2023).

AI has proven invaluable in biomarker analysis, particularly during the COVID-19 pandemic. By analyzing omics and clinical datasets, AI and ML algorithms have enabled effective patient stratification and management. These tools have identified critical biomarkers indicating COVID-19 severity and survival, assisting clinicians in prioritizing treatments for patients. Additionally, AI-driven analyses have uncovered gene networks associated with disease severity, underscoring the importance of clinical biomarkers in predicting disease progression (Bello et al., 2023).

## AI in metabolomics

Metabolomics has diverse applications, including analyzing metabolic products of the human gut microbiome, identifying biomarkers for disease diagnosis, prognosis, and monitoring, supporting cancer research by uncovering biomarkers and metabolic pathways, aiding studies on neurodegenerative diseases such as Alzheimer’s and Parkinson’s, investigating xenobiotic exposures, discovering new drug candidates, and studying drug metabolism, toxicology, efficacy, and potential side effects. Additionally, metabolomics integrates with other omics domains to provide a comprehensive view of biological systems (Chi et al., 2024). However, metabolomics generates vast datasets comprising hundreds to thousands of metabolites. Incorporating AI into metabolomics provides deeper insights into metabolic networks, advancing diagnosis, prognosis, and personalized treatment approaches for various diseases. ML techniques, including **decision trees, deep learning (DL), neural networks (NN), random forests (RF), and support vector machines (SVM)**, are employed to classify, regress, or cluster complex metabolomic data (Galal et al., 2022).

Recently, ML techniques have been applied to metabolomics data from various diseases, offering significant insights into metabolic profiles. In cancer research, AI is used to identify metabolic signatures, develop predictive models for cancer detection, prognosis, and recurrence, and analyze metabolomic data to uncover potential biomarkers and metabolic pathways. These approaches have been applied across multiple cancer types, including ovarian, breast, endometrial, hepatocellular carcinoma, gastric cancer, lung, squamous cell carcinoma, non-Hodgkin’s lymphoma, renal cell carcinoma, and osteosarcoma (Galal et al., 2022; Chen et al., 2024). In noncancer conditions, AI analyzes metabolomic data to identify biomarkers, disease signatures, and predictive models for various diseases, such as COVID-19, type 2 diabetes, nonalcoholic fatty liver disease (NAFLD), acute myocardial ischemia (AMI), chronic kidney disease (CKD), celiac disease, multiple sclerosis (MS), major depressive disorder, schizophrenia, and autism spectrum disorders. Additionally, AI applications extend to areas such as determining gestational age (Galal et al., 2022).

## AI and cancer diagnosis and prediction

AI tools, including ML and deep learning (DL), are revolutionizing cancer diagnosis by enhancing accuracy, improving efficiency, and offering noninvasive techniques. By analyzing medical data such as genomic sequences, imaging, and electronic health records, AI aids in identifying early-stage cancer biomarkers, improving recovery rates, and reducing mortality. These technologies are becoming integral to oncology and preventive healthcare. Integrating AI with genomic studies helps identify cancer-related genes, supporting precision medicine tailored to patients’ genetic profiles.

In addition to AI, technologies like big data analytics, cloud computing, and the Internet of Things (IoT) play vital roles in early cancer detection. Big data enables the analysis of large, complex datasets to uncover early cancer indicators, while cloud computing provides secure and efficient platforms for managing vast medical data. Wearable sensors collect biomarker data, offering real-time updates on potential cancer developments.

Advanced AI tools are improving the accuracy of cancer diagnosis through methods such as mammography and CT scans, often outperforming traditional techniques in detecting cancers like breast and lung cancer. AI has shown significant promise in enhancing breast cancer screening by identifying additional cases, improving positive predictive value, and reducing unnecessary recalls (Ng et al., 2023). For instance, the AI tool **Mia**, developed by Imperial College London and Kheiron Medical Technologies, was found to detect 13% more early-stage breast cancers (Transforming Cancer Diagnostics, 2024). Mia detected more cancers and led to more women being recalled, according to a release from Imperial College London. Mia was named one of the biggest seven medical breakthroughs in 2023 by ABC News (7 of the biggest medical breakthroughs in 2023, 2024). In a recent evaluation by Britain’s National Health Service, Mia analyzed mammograms from over 10,000 women. Mia accurately identified patients who had cancer, including 11 cases that were initially missed by human doctors. Mia’s impact underscores AI’s potential to enhance diagnostic accuracy in breast cancer screening (NHS AI Test, 2024). AI platforms such as **CHIEF** (Wang et al., 2024), **Sybil** (Aro et al., 2024), **IBM Watson for Oncology** (Jie et al., 2021), and **Tempus** (2024) are further transforming cancer diagnosis and prediction by facilitating early detection, personalized treatment plans, and improved patient outcomes. These platforms significantly reduce diagnosis time while meticulously analyzing imaging and genomic data (Arefin, 2024). An AI model achieved 96% accuracy in diagnosing invasive lobular carcinoma (ILC) using genetic mutations as ground truth (Pareja et al., 2024). In prostate cancer, AI systems have matched the diagnostic capabilities of experienced physicians, enabling early and accurate detection (Shucai and Heyuan, 2024). AI is also revolutionizing colorectal cancer (CRC) diagnosis and treatment, with advancements in classification, detection, digital pathology, endoscopic data processing, high-precision medical image analysis, personalized treatment, and robot-assisted surgery. These developments have substantially improved diagnostic accuracy for CRC. Machine learning prediction models enable faster and more accurate earlystage diagnoses, increase treatment success rates, and reduce colorectal cancer mortality. Furthermore, AI optimizes the allocation and utilization of medical resources, enhancing healthcare efficiency (Sun et al., 2024).

## AI in biomedical image analysis and digital pathology

Recently, a team of scientists has created **Biomed-Parse**, an AI model designed for medical image analysis. BiomedParse can handle nine imaging modalities, enhancing the prediction of systemic diseases by unifying segmentation, detection, and recognition tasks. The model introduces new capabilities, such as segmenting objects through textual descriptions, significantly improving accuracy and expanding applications. To develop BiomedParse, researchers created a dataset of over 6 million triples, including images, segmentation masks, and textual descriptions, using natural language labels from existing datasets. By integrating object recognition, detection, and segmentation, **BiomedParse** outperforms existing tools like **MedSAM** (Ma et al., 2024) and **SAM** (2024), particularly in handling irregularly shaped objects. Its ability to segment and label all objects in an image simultaneously makes it a comprehensive tool for biomedical image analysis, paving the way for efficient and accurate discoveries (Zhao et al., 2024).

Another groundbreaking AI tool is **Prov-GigaPath**, designed to address the unique computational challenges of digital pathology. Gigapixel slides, comprising tens of thousands of image tiles, require advanced models to process their immense size effectively. Prov-GigaPath is an open-access, whole-slide pathology foundation model pretrained on over one billion 256 × 256 pathology image tiles from more than 170,000 whole slides. By incorporating pathology reports into visionlanguage pretraining, Prov-GigaPath integrates realworld data and supports comprehensive slide modeling. Prov-GigaPath stands out as an open-weight model, excelling in various digital pathology tasks and paving the way for advancements in biomedical discoveries (GigaPath, 2024; Xu et al., 2024).

## AI in automated cell tracking and microscopy

Microscopic image analysis is a cornerstone of biological research. AI, particularly deep learning algorithms, has revolutionized this field by enabling faster and more accurate identification of cells and tissue structures. AI is revolutionizing the field of microscopy by enhancing the detection and classification of cells through advanced algorithms. Recently, **DeepTrack 2.0** was introduced, a user-friendly software that simplifies the creation, training, and validation of deep-learning models for digital microscopy. DeepTrack 2.0 supports applications such as particle tracking and cell classification, making deep-learning-enhanced video microscopy more accessible to a broader audience (Midtvedt et al., 2021). Additionally, AI algorithms enable realtime microscopy image analysis, facilitating immediate decision-making during image acquisition. Researchers utilized artificial intelligence for real-time cell detection and classification in automated microscopy, employing a high-dimensional feature extractor and machine learning, particularly random forests, to enhance execution performance while maintaining accuracy in biological image analysis (Balluet et al., 2022). AI has also automated cellular segmentation in microscopy images (Eisenstein et al., 2023), improving the accuracy of cell detection and enabling the extraction of quantifiable cellular features. These advancements are critical for understanding cellular organization in various pathologies (Durkee et al., 2021). A paper from 2020 presents a neural architecture search (NAS) method developed to optimize network designs for cell segmentation, achieving better performance compared to traditional methods (Zhu and Meijering, 2020).

Automated cell tracking has become an invaluable tool in biological research. Advances in optical microscopy and machine learning, especially deep neural networks, have driven the need for improved tracking algorithms. The **Cell Tracking Challenge (CTC, http://celltrackingchallenge.net)** was created to promote the development and evaluation of these algorithms. Since its launch in 2013, the CTC has offered a free repository of annotated microscopy videos and standardized evaluation metrics. AI techniques, including deep learning models like **U-Net**, **HRNet, R-CNN**, and **recurrent neural networks**, have significantly advanced cell tracking, improving accuracy and enabling more insightful research (Maška et al., 2023).

## AI and spatial omics

Spatial omics is a transformative field focused on measuring and mapping biomolecules such as RNA, DNA, and proteins directly within their native tissue environments. This innovative approach enables researchers to observe the spatial organization and interactions of cells, offering unprecedented insights into cellular functions and disease mechanisms. A critical step in spatial omics image analysis is cell segmentation, which facilitates the accurate identification and isolation of individual cells within tissue samples. By preserving spatial context, segmentation allows precise molecular analysis and a deeper understanding of cellular processes. This combination enables highly precise localization and quantification of biomolecules within tissues, allowing for a nuanced view of cellular behavior and interactions. The integration of spatial omics with advanced AI technologies is transforming the landscape of biological research, offering novel ways to explore the complexities of how cells interact and the impact these interactions have on health and disease. This synergy between spatial omics and AI not only enhances our understanding but also opens up new possibilities for diagnostics and therapeutic strategies. Scientists at the Children’s Hospital of Philadelphia (CHOP) have recently announced the creation of an AI tool called **CelloType.** CelloType is open-source software available in a public repository for noncommercial use (CelloType, 2024). This model was designed to more accurately identify and classify cells in high-content tissue images (Pang et al., 2024). Another similar AI tool is **NVIDIA VISTA-2D**. This model advances cell segmentation and morphology analysis by leveraging NVIDIA’s deep learning technology. It is designed to handle large-scale tasks and improve the accuracy of cell detection and segmentation (NVIDIA DEVELOPER, 2024). Two companies, NanoString Technologies and NVIDIA have joined forces to advance spatial biology research. **NanoString’s CosMx Spatial Molecular Imager** (**SMI**) platform integrates NVIDIA’s GPU technology, enabling the rapid processing and analysis of large datasets. This collaboration allows for the imaging of the entire transcriptome within cells and tissues, providing deeper insights into cellular functions and disease mechanisms (NVIDIA, 2024a).

AI has also facilitated advancements in 3D cell detection and segmentation. Researchers trained an object detector called **CellFinder** for automated 3D cell detection in large-scale images, including neuronal somata in whole-brain images of mice (Tyson et al., 2021; BrainGlobe, 2024). In 2024, the **CellSAM** tool was introduced, excelling in segmenting images of mammalian cells, yeast, and bacteria (Israel et al., 2024) across various imaging modalities. CellSAM builds upon CellFinder by retraining it to prompt the **Segment Anything Model (SAM)** for segmentation. This innovative approach enables a single AI model to accurately segment images of mammalian cells in tissues and cell culture, as well as yeast and bacteria, collected across various imaging modalities (CellSAM, 2024).

## AI and electron microscopy (EM)

Recent advancements in EM have been significantly enhanced by ML-based analysis. Research by Xu et al. (2021) demonstrates the integration of ML techniques in improving focused ion beam-scanning electron microscopy (FIB-SEM). These advancements include enhanced data acquisition, improved signal detection, and faster scanning, enabling the imaging of cellular samples at nanometer resolution. ML-based analysis efficiently processes and interprets complex imaging data, providing detailed visualizations of cellular structures.

Similarly, Heinrich et al. (2021) have made strides in data acquisition and ML-based analysis in microscopy, improving the ability to map and analyze molecular interactions within cells. These developments bring researchers closer to a comprehensive understanding of cellular physiology. Collectively, these studies highlight the use of AI and ML in overcoming traditional microscopy limitations and advancing the field of cell biology (Swedlow and Collinson, 2021).

## AI and infectious diseases

AI has made significant advancements in infectious diseases, playing a crucial role in developing diagnostic and therapeutic methods, forecasting outbreaks, optimizing treatment plans, and analyzing diagnostic images. It is instrumental in discovering anti-infective drugs and vaccines and combating the rise of antimicrobial resistance.

AI-powered clinical decision support systems forecast disease outbreaks, assist with precise diagnoses, enhance treatment plans, and track epidemiological trends by analyzing extensive datasets. Furthermore, AI improves the analysis of diagnostic images and disease identification, enabling quicker and more accurate results. By examining large datasets, AI systems boost diagnostic precision and treatment strategies, predict disease outbreaks, and monitor epidemiological patterns (Aslan, n.d.). Advances in AI applications for infectious diseases hold promise for more effective intervention strategies and improved public health protection (Aslan, n.d.; Singh, 2024).

For instance, AI was used to predict COVID-19 hospitalization and mortality. While these techniques show promise, further validation is required to address related challenges (Shakibfar et al., 2023a). Another study highlighted AI’s potential in creating a Disease Risk Score for predicting COVID-19 hospitalization and mortality using health registry data. This approach demonstrates AI’s ability to identify high-risk individuals, though it faces issues with generalizability and external validation (Shakibfar et al., 2023b).

Hien et al. (2024) utilized real-world clinical data to develop predictive models for severe COVID-19 outcomes, demonstrating the significant role of machine learning (ML) in the early identification and management of high-risk patients. Similarly, Banoei and colleagues employed ML techniques to forecast mortality among hospitalized COVID-19 patients. Their analysis of relationships between various risk factors identified critical indicators such as low oxygen levels and chronic kidney disease. This study provides a foundational understanding of these risk interactions, aiding in the prioritization of treatment approaches (Banoei et al., 2023).

Expanding beyond COVID-19, Gao et al. (2023) analyzed risk factors and predictive models for pulmonary tuberculosis, introducing **TBINet**, a deep-learning model leveraging CT images to identify pulmonary tuberculosis (PTB) infectivity. Validated by high AUC values and gradient-weighted class activation mapping (Grad-CAM) technology, this approach offers a swift and reliable diagnostic method, showcasing the utility of AI in medical imaging analysis for tuberculosis diagnosis and control. Their study demonstrated the feasibility of using CT images to rapidly and cost-effectively identify PTB infectivity through deep-learning methods.

AI has also been effectively employed in diagnosing Hansen’s disease (HD). It excels in rapid case detection, personalized treatment planning, mental health counseling, case classification, ensuring compliance with multidrug therapies, tracking geographical treatment distribution, and identifying adverse drug reactions and antimicrobial resistance. Moreover, AI plays a pivotal role in the early detection of nerve damage, which is crucial for preventing disabilities and planning rehabilitation. These capabilities are especially valuable in regions with a shortage of trained healthcare professionals (Deps et al., 2024).

The integration of AI into the management of infectious diseases holds immense potential to revolutionize diagnosis, treatment, and understanding of disease mechanisms. AI has proven to be highly effective in predicting, detecting, and controlling the spread of infectious diseases, as particularly highlighted during the COVID-19 pandemic. This technology plays a critical role in preventing future health crises by predicting outbreaks, identifying high-risk areas, and aiding vaccine development. Additionally, AI’s ability to track and trace infected individuals, identify potential hotspots, limit the spread of infections, and monitor patient symptoms empowers healthcare professionals to deliver more effective treatments (Siddig et al., 2023; Hsu et al., 2024).

## AI and vaccine design

AI technology has significantly enhanced the vaccine development process, providing innovative solutions to accelerate and optimize vaccine design. Researchers at Baidu Research developed an AI tool called **Linear Design**, which designed the optimal mRNA sequence for the SARS-CoV-2 spike protein in just 11 min. This break-through achieved a 128-fold increase in the COVID-19 vaccine’s antibody response and significantly improved vaccine stability (Dolgin, 2023; Zhang et al., 2023).

AI is also revolutionizing bacterial vaccine development. AI tools have identified 22 putative antigens for *Helicobacter pylori*, characterized T-cell epitopes for *Mycobacterium*, and validated the membrane protein FilF as a potential vaccine candidate for *Acinetobacter baumannii*. Additionally, AI is aiding vaccine development for *Klebsiella pneumoniae*, *Pseudomonas aeruginosa*, and *Streptococcus pneumoniae* (Gorki and Medhi, 2024). The potential of AI extends to parasitic infections, such as gastrointestinal nematodes (GINs), where AI techniques are being applied to develop effective anti-GIN vaccines. By increasing precision, accelerating design processes, and expanding our understanding of disease mechanisms, AI is transforming vaccine production. Despite these advancements, rigorous laboratory testing and regulatory approval remain essential to ensure vaccine safety and efficacy.

## AI models support scientific research

Starting in July 2024, scientists at Los Alamos National Laboratory in New Mexico began evaluating the **multi-modal LLM model GPT-4o** (2024) in real-world laboratory settings. This groundbreaking project aims to assess GPT-4o’s ability to assist both expert and novice scientists with complex biological tasks through its visual and voice capabilities. Tasks include transformation (introducing foreign genetic material into a host organism), cell culture (maintaining and propagating cells *in vitro*), and cell separation (e.g., through centrifugation). By integrating AI into standard laboratory workflows, the project seeks to drive innovation in biosciences while identifying potential risks associated with AI-assisted research (OpenAI and Los Alamos National Laboratory, 2024; Pannu et al., 2024). The potential of AI-assisted biological research to enhance human health and well-being is clear, yet significant unpredictabilities and risks remain. Researchers have emphasized the need for swift government action to establish comprehensive testing and mitigation strategies, leveraging decades of expertise to address large-scale biological research risks (Pannu et al., 2024). In 2023, Microsoft conducted evaluations of **GPT-4**, revealing its ability to provide detailed guidance for using the Rosetta protein design tool. This tool successfully created an antibody capable of binding to the SARS-CoV-2 spike protein. GPT-4 also demonstrated capabilities in automating biological experiments by converting experimental protocols into code for liquid-handling robots, significantly expediting laboratory workflows (Microsoft Research AI4Science, 2023). Further advancements were demonstrated by researchers at Carnegie Mellon University, who developed **Coscientist**, a system powered by GPT-4 that could design, plan, and execute complex experiments, including chemical syntheses. This system was capable of searching through documents, writing code, and operating robotic lab devices (Boiko et al., 2023). Recently, researchers from Stanford University and the Chan Zuckerberg Biohub introduced a **Virtual Lab**. This system comprises a group of large language model agents powered by GPT-4o that managed to design effective SARS-CoV-2 nanobodies (a type of antibody) with minimal human intervention (Swanson et al., 2024).

## AI in drug repurposing and discovery

AI presents an exciting future for rapid drug repurposing and discovery. By analyzing vast biological and chemical datasets, AI uncovers hidden connections between existing drugs, disease targets, and potential new treatments (Singh, 2024). AI’s capability to examine large datasets of drugs and disease targets is a potent tool for discovering new therapeutic uses for existing medications (Singh, 2024). By analyzing extensive biological and chemical datasets, AI can reveal hidden links between existing drugs, disease targets, and new therapeutic uses.

For example, Baricitinib, initially developed for rheumatoid arthritis, has shown potential as a COVID-19 treatment due to its anti-inflammatory and antiviral properties. AI analysis of Baricitinib’s interactions helped researchers predict and manage potential side effects, enabling safer clinical trials (Cantini et al., 2020; Stebbing et al., 2020; Saber-Ayad et al., 2021; Richardson et al., 2022). Similarly, Lopinavir/Ritonavir, an HIV medication, was investigated for its ability to inhibit a crucial SARS-CoV-2 enzyme (protease). However, AI analysis identified potential adverse effects on liver function, highlighting the drug’s limitations. This insight emphasizes the need to develop new, targeted inhibitors specifically designed to combat SARS-CoV-2, potentially overcoming the constraints of repurposed drugs (Parvathaneni and Gupta, 2020; Singh, 2024).

By analyzing chemical structures and predicting their interactions with disease targets, AI can uncover potential candidates across various therapeutic fields. For instance, Alendronate, commonly used to treat osteoporosis, exemplifies the efficacy of AI in drug repurposing. AI identified its ability to inhibit a key enzyme essential for the proliferation of certain cancer cells, suggesting its potential as a cancer treatment and broadening the spectrum of candidate drugs beyond traditional considerations (Saul and Einav, 2020; Alachram et al., 2021; Usha et al., 2021; Singh, 2024).

AI has become an invaluable tool, leveraging extensive datasets to forecast and refine drug characteristics, resulting in safer and more efficient repurposed medications. Current efforts include using AI to optimize dosing schedules of antiretroviral drugs for HIV, enhancing patient adherence and minimizing side effects (Xing et al., 2020; Serghini et al., 2023).

In the pharmaceutical industry, AI addresses significant challenges by detecting biological activity in preclinical screenings, optimizing pharmacokinetic properties for better formulations, predicting early toxicity to reduce attrition rates, and proactively screening for genetic mutations in biological targets to prolong therapeutic effectiveness (Serghini et al., 2023). Furthermore, integrating AI with patent data analysis offers a robust approach to identifying and repurposing existing drugs for new uses. This accelerates the development of costeffective, accessible treatments and enhances the healthcare system’s preparedness for emerging diseases.

By examining expired patents, AI can identify drugs no longer under patent protection, making them available for repurposing. This approach is particularly beneficial for antiviral treatments targeting less common viruses, where developing new drugs may not be economically feasible. It accelerates drug repurposing and ensures more efficient use of resources in addressing pathogenic threats. For instance, AI analysis identified Teicoplanin, an antibiotic no longer under patent protection, as having potential antiviral properties against the Zika virus (Dalal and Biswas, 2024; Ishaq et al., 2024).

The integration of computational and experimental methods is critical for discovering and developing molecules to combat deadly diseases. Computational approaches include active site prediction, homology modeling, ligand preparation, molecular dynamics simulation, molecular docking, pharmacophore modeling, target identification, and virtual screening. These AI-enabled capabilities highlight the potential of computer-aided drug design (CADD) to streamline and enhance the drug discovery process (Dalal and Biswas, 2024).

AI also plays a pivotal role in designing clinical trials for repurposed drugs. It can optimize dosages, identify outcome measures tailored to the drug and targeted virus, select highly relevant patient groups most likely to benefit, and determine treatment durations. This approach reduces costs, resource needs, and time to market while enabling researchers to achieve more conclusive trial results (Chopra et al., 2023).

The application of AI in drug development represents a significant breakthrough, enabling more efficient and effective discovery of medications, particularly for chronic diseases. By streamlining the drug discovery process, reducing costs, and accelerating the timeline for bringing new treatments to market, AI has the potential to revolutionize the pharmaceutical industry and improve healthcare outcomes.

## AI and biosimulation

Biosimulation, which involves simulating biological systems and processes using mathematical models, leverages AI algorithms for pattern recognition in clinical trials and the analysis of connections between drugs, patients, demographics, and trial parameters. These models empower researchers to address questions related to optimal dosing, medication interactions, and population-level efficacy.

For instance, **VeriSIM Life’s BIOiSIM** platform utilizes AI and ML to simulate the effects of chemicals on individual organs and entire bodily systems. This allows researchers to investigate optimal dosages, drug interactions, and overall effectiveness across populations while accelerating drug development and reducing reliance on extensive animal testing (BIOiSIM, 2024).

Other leaders in AI-driven biosimulation include **Certara**, which specializes in pharmacokinetic–pharmacodynamic (PK/PD) simulation and toxicokinetic (TK) modeling (Phoenix WinNonlin™ Software, 2024). Their **SimcypPBPK** platform is widely used to describe drug behavior in various body tissues, predict drug toxicity, and optimize dosing regimens (Simcyp™ PBPK Simulator, 2024).

**Simulations Plus** develops various models to predict drug toxicity and interactions, aiding research processes. Their **GastroPlus** software is an advanced simulation tool that models different types of drug absorption and pharmacokinetics in both humans and animals. It covers intravenous, oral, oral cavity, ocular, inhalation, dermal, subcutaneous, and intramuscular administration routes (Innovative science-based software, 2024). **Schrodinger** offers tools for molecular modeling and chemical engineering simulations, aiding in the discovery of new drugs (Opening New Worlds for Molecular Discovery, 2024). **Genedata** utilizes AI for data management and simulation in drug development processes, resulting in more efficient and cost-effective drug development (Digitalizing Biopharma R&D, 2024).

## AI in clinical trials

AI is revolutionizing clinical trials by speeding up data analysis, optimizing study designs, and streamlining patient recruitment, thereby boosting efficiency and cutting costs (Hutson, 2024). AI is employed in clinical studies to perform tasks such as data analysis, protocol preparation, and patient recruitment. It can help lower clinical trial drop-out rates, analyze videos to ensure medication adherence and answer patient questions through chatbots like **ChatDoctor** (Li et al., 2023). In a 2024 article published in *Nature* (Hutson, 2024), various AI platforms in clinical trials were discussed, such as **HINT**, which predicts trial success; **SPOT**, which analyzes trial timing; **SEETrials**, which extracts safety and efficacy information; **CliniDigest**, which summarizes clinical trial records; **Trial Pathfinder**, which assesses participation criteria; and **Criteria2Query**, which converts eligibility criteria into database queries. Additional platforms include **DQueST** for helping patients search for trials, **TrialGPT** for matching patients with trials, **Unlearn** for creating digital twins for control groups, **PLIP** for managing and organizing trial data, **AutoCriteria** for extracting eligibility criteria, **ChatTrial** for answering trial-related questions, and **SDQ** from **Saama**, which assists with data cleaning and milestone prediction.

Implementing AI in clinical trials faces challenges such as potential bias, difficulties in reproducing results, data privacy and security risks, over-reliance on AI, and the complexity of algorithms leading to a lack of transparency. Despite these challenges, the FDA recognizes the growing role of artificial intelligence and machine learning (AI/ML) throughout the drug development cycle and their potential to accelerate the process.

For instance, AI/ML methods can assist in clinical trials by selecting patients based on baseline characteristics such as demographic data, clinical information, vital signs, laboratory results, medical imaging, and genetic data to predict clinical outcomes following investigational treatments. These predictive models can identify patients with worse prognoses or those most likely to benefit from a treatment, ultimately aiding in demonstrating a drug’s effectiveness.

On November 8, 2022, the FDA issued an Emergency Use Authorization (EUA) for anakinra (Kineret) to treat COVID-19 in hospitalized adults with pneumonia requiring supplemental oxygen (lowor high-flow) who were at risk of progressing to severe respiratory failure (SRF) and likely to have elevated levels of plasma soluble urokinase plasminogen activator receptor (suPAR). Elevated suPAR levels are indicative of increased inflammation or immune response (Winnicki et al., 2019). Anakinra is the first interleukin-1 inhibitor authorized for COVID-19 treatment.

Notably, the FDA developed an in silico scoring rule as an alternative method to identify suitable patients for anakinra treatment in the absence of a commercially available suPAR assay in the United States. This scoring rule utilized AI/ML to analyze clinical characteristics and laboratory tests to predict patients likely to have elevated suPAR levels, a key criterion for anakinra treatment. This marked the FDA’s first use of AI/ML to identify an appropriate patient population for drug therapy (Liu et al., 2024).

## AI in early disease diagnosis

Hospitals and medical research institutions are increasingly developing AI tools for disease detection. These tools study and analyze symptoms, medical histories, and diagnostic processes to identify whether a patient is at risk for or experiencing the early stages of a disease. Early intervention and therapy facilitated by detection algorithms can slow disease progression or alleviate symptoms. Contract Research Organizations (CROs) may use these algorithms to identify and enroll patients at earlier stages of disease development, particularly during the prodromal phase. **IQVIA** has developed a data-driven illness detection program that evaluates a patient’s symptoms and characteristics, provides treatment recommendations—including clinical trials—and makes expert referrals (Leveraging Real World Data to Measure Disease Severity, 2024). Other companies leading in AI-driven disease detection include **AiCure**, which enhances treatment through real-time patient monitoring and data analysis (Patient Engagement, 2024); **United Imaging,** which offers AI-based solutions to improve diagnostic imaging accuracy (Alabama gets its first uCT^®^ ATLAS, 2024); and **Ibex Medical Analytics,** which provides AI solutions for pathology analysis (Trusted Cancer Diagnostics, 2024).

## AI and small molecule drugs

AI tools can analyze extensive datasets of existing drugs and drug candidates to uncover groundbreaking new insights. Artificial intelligence systems can forecast interactions between small molecule drugs and their target proteins, predict potential side effects, identify relationships and patterns within drug datasets, and enable researchers to develop new small molecule drugs with improved pharmacokinetic profiles, minimized side effects, and enhanced efficacy (Kirkpatrick, 2022). Recent advancements underscore how AI is revolutionizing the design and discovery of small molecules, with examples including USP1 inhibitors, KAT6A inhibitors, INS018_055, and small-molecule candidates in oncology, immunology, neuroinflammation, neurology, and cardiometabolic diseases (Dealmakers, n.d.).

## AI voice assistants

AI-driven voice assistants are increasingly being employed in clinical trials to manage various routine monitoring tasks, such as reminding patients of upcoming appointments and tracking their daily activities. AI voice assistants like Alexa, Google Assistant, and Siri have the potential to transform healthcare by turning speech into a valuable health indicator, enabling the early detection and prediction of potential health conditions. Specific acoustic features in speech have been linked to psychiatric disorders such as depression, PTSD, anxiety, and eating disorders (Low et al., 2020). For example, a vocal biomarker has been associated with hospitalization and mortality rates in patients with heart failure (Maor et al., 2020), and vocal biomarkers have been shown to correlate with depression severity and treatment response (Mundt et al., 2012). By capturing and analyzing subtle voice changes, AI can generate a range of health measurements to provide a more comprehensive picture of overall health. Machine learning technology using speech samples, obtained either in clinical settings or remotely, could eventually serve as a biomarker to improve diagnosis and treatment. Current research primarily focuses on using speech’s acoustic features to detect conditions like depression and schizophrenia (Low and Bentley, 2020).

## Companies engaged in AI/ML-enabled discovery

Over the years, the interest in applying AI to drug R&D has significantly increased, driven by expectations of faster timelines, reduced costs, and the ability to uncover hidden insights from large datasets. More than 150 AI-focused companies have raised substantial funding, particularly in small-molecule drug discovery, through venture capital financings, initial public offerings (IPOs), and high-value partnerships with large pharmaceutical companies (Table 1). Currently, the first AI-based small-molecule drug candidates have entered clinical trials (Kirkpatrick, 2022).

**Table 1:** AI-driven innovations in biotechnology: companies and their key products

Company (Ref.)	AI application area	AI technology	Partnerships/Collaborations
BenevolentAI (Kirkpatrick, 2022; Chopra et al., 2023)	Drug discovery, CKD and IPF, treatment, heart failure, and lupus research	AI-driven drug discovery	Partnership with AstraZeneca
Verge Genomics (Kirkpatrick, 2022)	ALS, neurodegenerative diseases	AI platform for target identification in neurodegenerative diseases	Partnership with Eli Lilly
NVIDIA Corporation (2024)	Medical imaging, genomics, and AI model development in healthcare and life sciences; treatment, diagnosis, and drug discovery	Clara Holoscan MGX	
Insitro (2024); Making Medicines (2024)	Drug discovery, neurodegenerative diseases, ALS and FTD treatment	Machine learning combined with high-throughput biology	Collaboration with Bristol Myers Squibb
Recursion Pharmaceuticals (Kirkpatrick, 2022)	Drug discovery, oncology, neuroscience	AI-guided drug discovery with image-based profiling of cellular disease models	Collaboration with Genentech, Roche
Exscientia (Kirkpatrick, 2022)	Drug discovery, oncology, immunology	AI-driven small-molecule drug design	Collaboration with Bristol Myers Squibb, Sanofi
Insilico Medicine (Kirkpatrick, 2022)	Drug discovery, immuno-oncology	AI-driven preclinical research, small-molecule drug development; ISM004-1057D (a first-in-class small-molecule inhibitor of the enzyme QPCTL regulating the CD47–SIRP pathway)	Partnership with Fosun Pharma
Relay Therapeutics (Kirkpatrick, 2022)	Cancer, drug discovery	Drug discovery, RLY-1971 (SHP2 inhibitor development for cancer)	Collaboration with Genentech
Takeda Pharmaceuticals (Merck, 2024; Prometheus Biosciences, 2024; Takeda, 2024)	Drug development, oncology, IBD treatment	Bioinformatics and machine learning for medical treatments and drug innovation; TAK-733 (REC-4881), a MEK inhibitor for hereditary cancer syndrome	Partnerships with Prometheus Biosciences, Recursion
ReviveMed’s AI (2024)	Cancer immunotherapies	AI platform	Bristol Myers Squibb
Sensyne Health (2024)	Study of myeloproliferative neoplasms development	Machine learning	Bristol Myers Squibb
Medidata (2024a, 2024b)	Clinical trials, drug development	AI-powered solutions for drug research and clinical trials	Adopted across the pharmaceutical, biotech, academic, government sectors, hospitals and clinical research organizations (CROs)
Saama Technologies (2024)	Clinical research, drug discovery	AI platform	
Health (formerly Pillsy 2024)	Healthcare	Mobile app for medication compliance monitoring and patient-reported information	
AliveCor (2024)	Cardiology, healthcare	Wearable EKG device for cardiac rhythm detection	Partnership with Medable

**BenevolentAI**, an AI-enabled drug discovery company, utilizes a knowledge graph that integrates publicly available biomedical and chemical data with proprietary datasets, allowing AI tools to generate target hypotheses. It focuses on identifying targets for chronic kidney disease (CKD), idiopathic pulmonary fibrosis (IPF), heart failure and systemic lupus erythematosus (Kirkpatrick, 2022; Chopra et al., 2023). **Verge’s** AI platform using a proprietary collection of patient brain transcriptomes, identifies targets for amyotrophic lateral sclerosis (ALS) and other neurodegenerative diseases (Kirkpatrick, 2022). **NVIDIA Corporation** launched Clara Holoscan MGX to enable the creation and deployment of real-time AI applications. Clara offers tools for medical imaging, genomics, and AI model development (NVIDIA, 2024b). **Insitro** combines machine learning with high-throughput biology. It generates biological datasets from cellular disease models, integrating them with human clinical data to identify therapeutic targets. Insitro aims to discover novel targets for ALS and fronto-temporal dementia (FTD) (Insitro, 2024; Making Medicines, 2024). **Recursion Pharmaceuticals** focuses on generating data from cellular models employing ML. By using image-based profiling of cellular disease models treated with various potential drug leads, Recursion identifies novel targets and medicines in neuroscience and oncology (Kirkpatrick, 2022). **Exscientia** specializes in AI-driven small-molecule drug design, oncology and immunology (Kirkpatrick, 2022). **Insilico Medicine** focuses on AI-driven preclinical research. Insilico initiated a phase 1 trial of the small-molecule inhibitor ISM001-055 for idiopathic pulmonary fibrosis. Using its AI platform, both the target and drug candidate were identified, reducing the time from target discovery to phase 1 trial initiation to less than 30 months. Partnership with Fosun Pharma led to nomination of ISM0041057D as a first-in-class small-molecule inhibitor of the enzyme QPCTL regulating the CD47–SIRPα pathway (Kirkpatrick, 2022). **Relay Therapeutics** specializes in identifying drug candidates based on protein dynamics. The company’s SHP2 inhibitor, RLY-1971 is currently in phase 1 trials for cancer treatment (Kirkpatrick, 2022). **Takeda Pharmaceuticals** aims to develop therapies and diagnostics for inflammatory bowel disease (IBD) using advanced bioinformatics and machine learning tools (Prometheus Biosciences, 2024). Takeda explored over 60 indications for preclinical and clinical molecules, identifying new treatment options within 18 months. A key advancement includes the clinical development of TAK-733 (REC-4881), a MEK inhibitor for hereditary cancer syndrome (Takeda, 2024). **ReviveMed’s** AI platform focuses on the mechanisms of response and resistance to cancer immunotherapies (ReviveMed, 2024). **Sensyne Health** (2024) leverages ML for studying the development of myeloproliferative neoplasms, a group of rare blood diseases. **Medidata AI** (2024b) offers AI tools designed to accelerate drug development, reduce risks, lower costs, and enhance patient outcomes. By harnessing AI algorithms and ML, Medidata AI assists hospitals and clinical research organizations (CROs) in executing clinical trials. Launch Therapeutics has chosen Medidata AI Intelligent Trials to expedite the preparation of clinical trials. This analytics system uses AI to enhance trial design and execution through real-time performance measurements, predictive models, and forecasting capabilities (Medidata 2024a). **Saama** (2024) offers products to expedite clinical research and commercialization, by streamlining key phases of clinical research and automating labor-intensive steps through AI and big data analysis. **Health** has developed a mobile app aimed at improving medication compliance in real-time research. The app includes features like reminder notifications, dosage tracking, educational content, and patient-reported information collection for healthcare providers (Pillsy, 2024). **AliveCor** (2024) focuses on biometric data collection through its wearable electrocardiogram (EKG) device. Using ML, the device detects irregular cardiac rhythms, such as atrial fibrillation (AF), by analyzing real-time data. AliveCor partnered with Medable to facilitate remote clinical trials in cardiology.

## AI and biosafety and biosecurity risks

AI developers anticipate that combinations of artificial intelligence techniques, including automation technologies, LLMs, and robotics, will enable experiments—such as the manipulation, design, and synthesis of DNA, drug candidates, or toxins—with minimal human involvement. These advances have the potential to transform biomedical research, but they also pose significant biosafety and biosecurity risks (Urbina et al., 2022).

In response to these growing risks, various governments have implemented measures to address safety concerns associated with advanced AI models. In 2023, the US government secured voluntary commitments from 15 leading AI companies to better manage AI-related risks. Later that year, US President Joe Biden issued an Executive Order to ensure the safe, secure, and trustworthy development and deployment of artificial intelligence. This order mandates that companies must inform the government before launching models primarily trained on biological sequence data and requiring more than 10^23 computing operations (Pannu et al., 2024).

In November 2024, representatives from ten governments participated in the first meeting of the International Network of AI Safety Institutes in San Francisco, California (2024). France is set to host the AI Action Summit in Paris in February 2025 (Elysee, 2024; Pannu et al., 2024). Countries such as Canada, Japan, Singapore, the United Kingdom, and the United States have established government institutes focused on AI safety, creating standards and tools to manage risks. Australia, the European Union (which has set up a safety unit within its AI Office), France, Kenya, and South Korea are the founding members of the International Network of AI Safety Institutes.

In the absence of comprehensive government policies to address urgent risks and mitigation strategies, companies like Anthropic and OpenAI have implemented in-house evaluation protocols. These protocols include automated assessments, red teaming—where humans attempt to elicit harmful capabilities—and controlled trials that compare task performance with and without AI assistance (Pannu et al., 2024). However, these evaluations often focus narrowly on the potential for AI models to aid in the development of bioweapons.

Current evaluations also tend to concentrate on basic laboratory tasks. For instance, OpenAI’s tests with Los Alamos researchers assess capabilities that, while critical for beneficial research, could also be used to develop harmful agents, such as crop-destroying pathogens. Additionally, an underexplored concern is the interaction of multiple AI systems. While the US government has highlighted this issue, most companies test only individual models, overlooking the broader risks of combined system behavior (Pannu et al., 2024).

## Guidelines on the use of AI tools in research publications

Organizations such as COPE, WAME, and the JAMA Network have established guidelines to address the growing use of AI tools, including ChatGPT and Large Language Models, in publications (Authorship and AI tools, 2024):

1. AI tools cannot be listed as authors, as they cannot take responsibility for submitted work, manage conflicts of interest, or handle copyright agreements.
2. Authors using artificial intelligence must disclose their use in the Materials and Methods section, specifying how and which tool was used.
3. Authors remain fully responsible for the manuscript’s content, including sections produced using AI techniques.
4. Authors must ensure compliance with publication ethics.

Publishers have varying editorial policies regarding the use of AI. Most journals, such as those published by AGU (2024), Elsevier (2024), JAMA (2024), PLOS (2024), PNAS (2024), Sage (2024), Science (2024), Springer Nature (2024), and Taylor & Francis (2024), allow the use of AI tools provided their use is disclosed. According to a study published in December 2024, 78 medical journals have issued guidance on AI use in peer review. Of these, 46 journals explicitly forbid the use of AI, while 32 permit it under the condition that confidentiality is maintained and authorship rights are respected (Li et al., 2024).

## AI tools for streamlining scientific paper writing

Presented below are seven subsections highlighting various AI tools designed to assist with text-related tasks (Table 2). These tools are categorized based on their functionalities: literature review, content creation, citation management, proofreading and optimization, formatting, and originality verification. Each subsection illustrates how these tools can enhance the efficiency, accuracy, and quality of scientific work.

**Table 2:** AI tools for streamlining scientific paper writing

Purpose	AI Tools
Literature review	SciSpace (2024), Microsoft Copilot (2024), PDFgear Copilot (2024), Trinka (2024), ChatPDF (2024), Consensus (2024), Scite (2024), Research Rabbit (2024), Semantic Scholar (2024), Elicit (2024), Clarivate (2024)
Content creation	BioloGPT (2024), GPT-4 (2024), DeepAI Text Generator (2024)
Citation management	Trinka (2024), Semantic Scholar (2024), Scite (2024), Mendeley (2024), Zotero (2024), EndNote (2024)
Proofreading & optimization	Wordvice AI (2024), Grammarly (2024), Hemingway Editor (2024), QuillBot (2024), Underleaf (2024)
Text formatting	Cite This For Me (2024), Zotero (2024), Scribbr (2024), SciSpace (2024), and Underleaf (2024)
Originality verification	iThenticate (2024), Turnitin (2024), Copyscape (2024), Crossref Similarity Check (2024), ZeroGPT (2024), Originality.AI (2024), SciSpace AI Detector (2024), Content at Scale AI Detector (2024), GPT-2 Output Detector (2024)
Retraction verification	WithdrarXiv (2024)

### AI tools in literature review

AI tools like **SciSpace** (2024), **Microsoft Copilot** (2024), **PDFgear Copilot** (2024) enhance research efficiency by analyzing and summarizing scientific papers, detecting errors, and suggesting corrections, thereby significantly improving the quality and speed of research. **Trinka** (2024) refines grammar, style, and clarity for academic writing, while **ChatPDF** (2024) processes PDF documents to extract key details and create summaries, boosting research efficiency and effectiveness. Another useful tool is **Consensus** (2024) that searches over 200 million scientific articles and provides features like the Consensus Meter, which indicates the general scientific agreement on a topic, helping identify the most relevant and reliable research papers. **Scite**’s (2024) database contains 200 million scholarly sources and over 1.2 billion citations. It evaluates references by showing the context of citations, assisting in assessing the impact and credibility of research. **Research Rabbit** (2024) is citation-based literature mapping tool that facilitates citation mining and organizes collections. Starting with “seed papers,” it automatically identifies additional relevant papers, visualizes networks of papers and co-authorships, and provides updates on new research. **Semantic Scholar** (2024) is a free AI tool for searching over 200 million academic papers. It generates summaries, highlights key and influential elements of papers, displays key citations, and analyzes articles. **Elicit** (2024) summarizes scientific articles from a database of 125 million academic papers, extracts details into organized tables, and synthesizes data for efficient research. **Clarivate** (2024) offers comprehensive solutions for literature reviews through its research database, **The Web of Science**. This subscription-based platform provides reference and citation data from academic journals and conference proceedings.

### AI tools useful in content creation

**BioloGPT** (2024) is a biology-focused AI that ensures accuracy by rigorously citing sources, generating new hypotheses, and addressing research biases with a critical and empirical approach. **GPT-4o** (2024) is OpenAI’s most advanced multimodal model, capable of efficiently generating text and excelling in non-English languages. **DeepAI Text Generator** (2024) offers multiple functions, including text generation, sentence completion, and contextually relevant content prediction, transforming input into coherent text.

### AI tools useful in citation management

For citation management, the AI tools such as **Trinka** (2024), **Semantic Scholar** (2024), and **Scite** (2024) mentioned in the “AI tools in literature review” section can be particularly useful. In addition to these, there are several other citation management tools like **Mendeley** (2024). This tool offers smart citation suggestions, research discovery capabilities, and personalized paper recommendations based on reading habits. It integrates with Elsevier journals for priority access to newly published papers and includes a social platform for researchers. **Zotero** (2024) is a popular open-source tool that integrates seamlessly with web browsers. It supports annotation, citation, collection, and organization of references, allowing users to create citations and bibliographies in various styles. Zotero also provides smart recommendations while browsing the web. **EndNote** (2024) is a robust tool for managing references and citations, offering features like intelligent citation matching and advanced collaboration tools for projects.

### AI tools useful in text proofreading and optimization

**Wordvice AI** (2024) enhances grammar, spelling, punctuation, and style. It also assists in paraphrasing to avoid plagiarism, translating text, and summarizing documents. **Grammarly** (2024) offers advanced grammar and style suggestions, making it particularly useful for improving the writing quality of scientific papers. **Hemingway Editor** (2024) improves text readability and simplifies language while maintaining clarity. **Quill-Bot** (2024) utilizes AI to assist users in enhancing, generating, and paraphrasing content. **Underleaf** (2024) specializes in correcting grammar, improving style, and rewording content tailored for academic writing.

### AI tools useful in text formatting

For formatting purposes, tools such as **Cite This For Me** (2024), **Zotero** (2024), and **Scribbr** (2024) can be particularly useful. These tools offer citation generators in various formats. Additionally, tools like **SciSpace** (2024) and **Underleaf** (2024) provide further assistance with formatting tasks.

### AI tools useful for originality verification

Ensuring originality and avoiding plagiarism is critical when writing a manuscript. It is also essential to verify that AI-generated content adheres to publication guidelines. Depending on the publisher, AI-generated text might be prohibited outright or require explicit labeling by authors during submission. Publishers often employ advanced tools like **iThenticate** (2024) to detect both plagiarism and AI-generated content. Other useful tools for detecting plagiarism and AI-generated text include: **Turnitin** (2024), **Copyscape** (2024), **Crossref Similarity Check** (2024), **ZeroGPT** (2024), **Originality**.**AI** (2024), **SciSpace AI Detector** (2024), **Content at Scale AI Detector** (2024), **GPT-2 Output Detector** (2024).

### AI tool useful for retraction verification

**WithdrarXiv** (2024) is the first dataset of withdrawn papers from arXiv, released in December 2024. It includes over 14,000 papers and their retraction comments, covering the repository’s history up to September 2024. This dataset is valuable for scientists, aiding in maintaining scientific integrity, improving quality control, developing automated verification systems, addressing ethical concerns, and serving as an educational resource for new researchers.

## AI’s risks and ethical challenges

AI holds the promise of transforming scientific and medical sectors enabling tasks that previously took years to be completed. Recent demonstrations have shown that AI-designed proteins can tackle the century-old issue of developing new treatments for snakebites, which claim around 100,000 lives each year (Callaway, 2025). However, despite these advancements, AI also poses significant risks and demands careful ethical considerations. Ensuring equitable access, fair distribution of AI technologies and their benefits, and high-quality healthcare services for all – irrespective of disability status, ethnicity, gender, geographic location, socioeconomic status, or race – is paramount. Addressing concerns about algorithmic fairness and biases, data privacy, ensuring informed consent for data usage, and maintaining safety and transparency is essential. Additionally, there are legal and social implications, as well as concerns regarding data security and confidentiality, the validity of research findings, and potential incidents of research misconduct, that must be resolved (Bouhouita-Guermech et al., 2023; Resnik and Hosseini, 2024).

To ensure AI benefits everyone fairly, several ethical challenges must be addressed: Mitigating biases in data collection and promoting diversity in research. Safeguarding privacy and confidentiality. Obtaining informed consent for the use of AI technologies. Ensuring human oversight in AI applications. Developing transparent AI systems. Clearly defining the roles and responsibilities of AI developers, health professionals, and institutions. This comprehensive strategy integrates human values into AI advancements, thereby enhancing public health and overall well-being (Health Equity and Ethical Considerations, 2024).

Beyond these ethical considerations, there are also risks, such as the potential introduction of errors by AI. Generative AI, including large language models (LLMs), are prone to hallucinations (Why scientists trust AI too much, 2025), although the exact mechanisms of the problem are not clear. These errors likely stem from a combination of factors such as data compression, ambiguities or mistakes in the AI’s training data, or incorrect facts or assumptions in prompts provided by users. To track this issue, the Hallucination Vulnerability Index was created, which sorts hallucinations into six categories and three degrees of severity. Additionally, the Hallucinations Leaderboard platform that tracks, ranks, and evaluates hallucinations in LLMs was launched (Jones, 2025). It was shown that some chatbots confabulate facts in up to 30% of cases, making up information that isn’t in the given document. The issue of false scientific references is particularly problematic. Study from 2024 demonstrates that various chatbots made errors in references between 30% and 90% of the time (Chelli et al., 2024). Thus, it is very important to verify generated information and perform external fact-checking.

Furthermore, other limitations of AI technology must also be considered. One notable example is Alpha-Fold 3, which has enhanced the precision of predicting biomolecular structures, yet still faces challenges with stereochemistry and requires human assistance (Steinkellner et al., 2025).

AI models for antimicrobial resistance diagnosis, discovery, and treatment often rely on imbalanced datasets, leading to potential reliability issues and representing only certain patient populations or specific biological tests. AI models developed using these biased datasets may struggle to generalize effectively beyond their specific training data (Cesaro et al., 2025).

It is crucial to critically evaluate AI’s role in research to prevent overdependence on its abilities. By addressing both ethical concerns and potential risks, while acknowledging the limitations of AI**,** the scientific community can leverage AI’s potential while maintaining research integrity.

## Conclusions

AI is transforming biomedical research by improving data analysis, modeling biological processes, and detecting diseases, leading to faster scientific discoveries. Scientists emphasize the usefulness of AI in summarizing scientific data from other researchers, accelerating administrative tasks, speeding up the process of writing scientific papers, generating new hypotheses, and conducting faster peer reviews. Despite its promising future, AI presents ethical challenges, including issues related to data quality, result interpretation, and responsible use. Addressing these challenges requires interdisciplinary collaboration and robust regulation. Ensuring the ethical use of AI – such as preventing plagiarism and maintaining content quality – is essential for safeguarding the integrity and progress of scientific research.
